# A Creutzfeldt‐Jakob disease case misdiagnosed with acute cerebral infarction and review of the literature

**DOI:** 10.1002/ccr3.3388

**Published:** 2020-10-19

**Authors:** Zhouwei Xu, Yuwu Zhao

**Affiliations:** ^1^ Department of Neurology Shanghai Jiao Tong University Affiliated Sixth People's Hospital Shanghai China

**Keywords:** Creutzfeldt‐Jakob disease, dizziness, mimics, neuroimaging, subtype

## Abstract

It was critical for the clinician to be aware of the neuroimaging and early‐onset symptoms of this fatal neurodegenerative disease, and avoid initiating inappropriate therapy. Neuroimaging plays a key role in differentiating it from other mimics.

## Graphical abstract

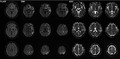

## 1 INTRODUCTION

Creutzfeldt‐Jakob disease (CJD) is a rare, fatal, and devastating neurodegenerative disease. The incidence rate of CJD ranged from 1.7 to 2.2 per million.^1^ The typical clinical manifestations include intellectual impairment, myoclonus, visual or cerebellar problems, pyramidal or extrapyramidal features, and akinetic mutism.^2^ The disease has a grave prognosis, and the life span ranges from several weeks to 1 year. Furthermore, there is no specific treatment for slowing down the progress of this disease. It is not well recognized and increasingly misdiagnosed due to various reasons, including variant manifestations and lack of available golden standard diagnostic tools in the clinical setting. Here, we report a case of probable sporadic CJD in a woman to raise the awareness of this prion‐transmitted infectious disease.

## 2 CASE REPORT

A 67‐year‐old woman presented with half a year history of dizziness. The dizziness worsened over the last 6 months, and the dizziness could be initiated by moving backward the neck. At the beginning of the disease onset, She had no other presentations, including vomiting, cognitive impairment, unconsciousness, and dysphagia. 6 months before admission, she was hospitalized at a local community hospital, and then, she was scanned with routine brain CT and neck MRI. The neck and brain imagings showed the disk herniation of the C1‐C2, C3‐C4, and some lacunar infarction in the bilateral basal ganglion, respectively. The local community hospital gave her routine anti‐platelet and antihyperlipidemic medications for the lacunar infarction.

**FIGURE 1:**
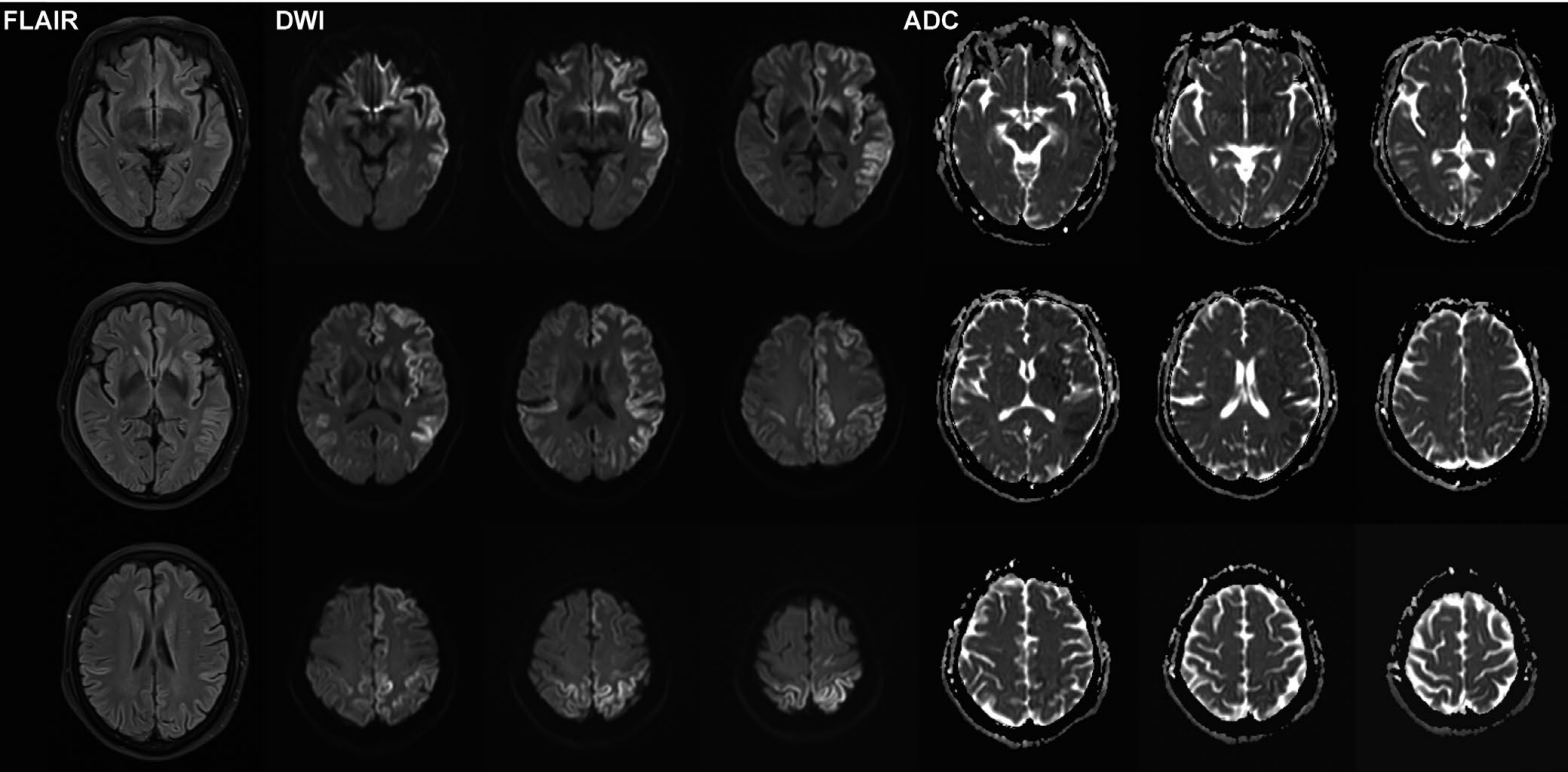
There was an increased signal intensity of the bilateral frontal‐temporal‐parietal cortex in the diffusion‐weighted imaging(DWI) sequence. In contrast, there was a decreased signal intensity of the counterpart cortex in the apparent diffusion coefficient (ADC) sequence. The signal hypointensity on the FLAIR sequence is less evident than the DWI sequence

After 4 weeks of treatment, the dizziness symptom did not improve, and the patient was then referred to the department of neurology of the Shanghai Sixth people's hospital for further neurological consultation. She complained the dizziness worsened over the last 2 months and weakness of her right lower limb. An additional brain MRI enhancement scan and brain CT angiography scan were performed. The MRI scan indicated that there was an increased signal intensity of the bilateral frontal‐temporal‐parietal cortex on the diffusion‐weighted imaging(DWI) sequence and brain atrophy (Figure 1). In contrast, there was a decreased signal intensity of the counterpart cortex on the apparent diffusion coefficient (ADC) sequence. Brain CT angiography showed normal. At that time, the doctor of the radiology department considered it as acute brain infarction. There were no abnormalities on her full blood test, renal, liver, thyroid function, erythrocyte sedimentation rate, and antinuclear antibody panel. The electrolyte examination showed that her blood potassium was 3.1 mmol/L (reference range 3.5‐5.5). The previous history revealed that she had half a year of diabetes, and she regularly took metformin to treat diabetes. She did not have a history of smoking and any other drug or alcohol overuse. She also had no significant family history of neurodegenerative diseases. The ultrasound showed there were plaques on her bilateral carotid arteries. The physical and neurological examination showed she had normal blood pressure and weakness of the right distal lower limb (Medical Research Council grade 4/5 ankle dorsiflexion and eversion and grade 4/5 ankle plantar flexion and inversion). The other tests were all within the normal range. Then, the patient was continued to be treated as acute cerebral ischemia with clopidogrel, atorvastatin, and butylphthalide to anti‐platelet and lower the level of cholesterol for the next 2 weeks. However, no remarkable improvement was observed during the period of hospitalization. The patient was discharged after 2 weeks of treatment.

**FIGURE 2:**
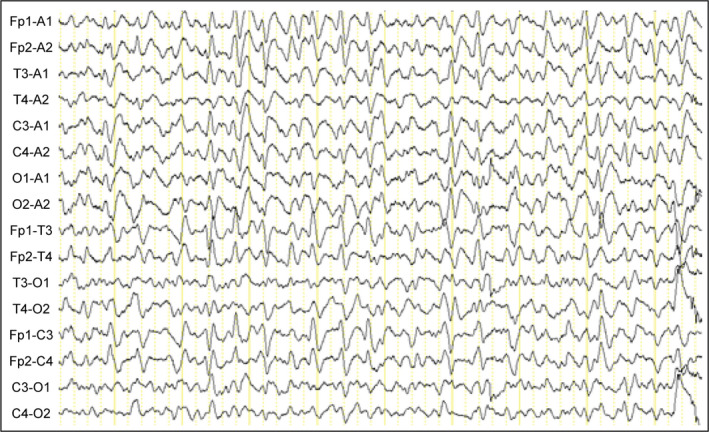
The synchronous triphasic sharp waves were seen in the frontal, temporal, occipital leads in the background of diffuse generalized theta‐to delta slow waves

Four months later, the patient experienced a hip fracture and underwent a hip replacement operation. After that, the patient was unable to ambulate and was confined to the bed at home. Meanwhile, her cognitive impairment progressed significantly and became fearful, nervous, and mute. The symptoms worsened progressively in the next 2 weeks. The patient was referred to our department again. The physical evaluation revealed she had startle‐related myoclonus, mutism, and hyperreflexia. The patient had little interaction with the family members, and had her eyes open but not tracking a face moving across the visual field. She could not cooperate with the Mini‐Mental State Examination. The patient sometimes moved and hold her bilateral upper extremities voluntarily in the air for no purpose. The electroencephalography (EEG) test was performed, and previous MRI images were reviewed. The EEG showed there were classical synchronous triphasic sharp waves over the bilateral frontal, temporal, occipital regions. (Figure 2). Creutzfeldt‐Jakob disease seems probable based on the new‐onset symptoms. However, the family member refuses to do the lumbar puncture for further CSF analysis to confirm the diagnosis. Due to the lack of pathological results, the patient was finally diagnosed as probable sporadic CJD based on the previous medical history, neurological findings, and MRI scan, which fulfilled the diagnostic criteria of sCJD.^2^ The patient was empirically treated for the muscle rigidity with eperisone hydrochloride and then transferred to the local center of disease control (CDC) to accept further treatment due to the infectivity of this disease.

## 3 DISCUSSION

Creutzfeldt‐Jakob disease is a prion‐transmitted disease, which comprises sporadic CJD, iatrogenic CJD, familial CJD, and variant CJD. sCJD accounts for 85%‐90% of the total cases.^1^ In the early stage of the disease, the clinical presentations are atypical and diverse, including vertigo, personality change, headache, cortical blindness, hallucination, seizure, and stroke‐like paralysis, which could mimic other conditions. In this case, the initial onset symptom is dizziness, which could masquerade as a posterior circulation stroke. The other posterior circulation symptoms, including vertigo, nystagmus, and cerebellar dysfunctions accompanying dizziness, were also investigated in the other studies. We undertook a review of the current literature, searching for reports in which dizziness was described as the first onset symptom on CJD patients (Table 1). Interestingly, with the progression of the disease, the typical manifestations gradually present within 4 weeks. The follow‐up period between first onset symptoms as dizziness and classical manifestations of CJD were also summarized in Table 1. The clinician should pay attention to these early‐onset symptoms, which are the key clues for considering the early stage of CJD, and further MRI scans and CSF investigation were required.

**Table 1:** Review of the published literature ( first onset symptom of CJD as dizziness)

Author	Sex	Age	First onset symptoms	Follow‐up period	Neuroimaging	EEG
Campbell et al^12^	Female	62	Dizziness, vomiting, headaches	4 wk	Several small, nonspecific, white‐matter lesions	Delta activity
Wei et al^13^	Male	41	Dizziness, vertigo	2 wk	Hyperintensity in occipital lobes	Occasional triphasic waves
Choi et al^14^	Male	66	Dizziness, gaze‐evoked nystagmus.	3 wk	High signal intensity in the nodulus, anterior cerebellum, front‐temporo‐occipital cortex, and basal ganglia.	Periodic sharp wave complexes
Dirzius et al^15^	Female	53	Dizziness, blurred vision, coordination impairment	2 wk	Slight increased signal intensity in the occipital cortex	Periodic sharp wave complexes in frontal regions
Torres Herrán et al^16^	Female	56	Dizziness, vertigo	4 wk	High signal abnormalities in caudate and putamen nucleus and a medial frontal and parietal cortical ribboning	Periodic sharp wave complexes
Xu et al^17^	Female	68	Dizziness, horizontal nystagmus, unconsciousness	1 wk	High signal intensity in the bilateral parietal occipital and frontal lobes	High amplitude of a triphasic sharp wave

Due to the variant symptoms of CJD, neuroimaging investigation is useful in making an early diagnosis of CJD when lacking the characteristic clinical manifestations. The imaging abnormalities could involve cingulate, striatum, thalamus, and neocortical cortex (precentral gyrus was always spared). One study showed that signal hyperintensity on the MRI DWI sequence had 96% sensitivity and 93% specificity for differentiating CJD from other rapid dementia diseases. The hyperintensity was more evident on DWI than FLAIR (Fluid‐attenuated inversion recovery), and the restricted diffusion was noted on ADC.^3^, ^4^ One study showed that a serial MRI scan within the disease course is necessary for reaching the diagnosis of CJD.^5^ Cortical DWI hyperintensity with normal ADC mapping has been reported in autoimmune encephalitis(AE), which is a key neuroimaging clue to distinct AE from sCJD.^6^ A PET‐CT scan is useful for improving diagnostic accuracy by showing a hypometabolic pattern in CJD patients.^7^ In this case, the classical ribboning lesion involved the patient's bilateral hemispheres, including frontal, temporal, occipital, and parietal lobes on the DWI sequence, which was consistent with the previous study.^3^ Pulvinar and hockey stick signs could be observed in 90% of the vCJD case and have 100% specificity for the diagnosis of vCJD.^8^ sCJD can be further classified into six subtypes MM1, MM2, MV1, MV2, VV1, and VV2, according to the different molecular strains. One study showed that the VV1 subtype is more vulnerable for the cortical involvement while sparing the basal ganglia and thalami.^9^ The relationship between MRI imaging and subtypes is still unclear and needs to be further investigated. Interestingly, the disease had little effect on this patient's motor function during its course, probably due to the precentral gyrus sparing.

Electroencephalography was a noninvasive assessment tool for the diagnosis of CJD. The typical EEG manifestations are the periodic triphasic sharp waves. Periodic sharp wave complexes (PSWC) can develop in half of the late stage of CJD patients.^10^ However, this EEG pattern was not specific to CJD and was also observed in other neurological diseases, including Lewy body dementia, metabolic encephalopathies, and nonconvulsive status epilepticus. Electroencephalography abnormalities were showed lower sensitivity and specificity in detecting the early stage of CJD.^8^

The CSF analysis also played a key role in the diagnosis of probable CJD. Several studies indicated that cerebral fluid real‐time quaking‐induced conversion (RT‐QuIC) of prion protein has a high specificity for CJD,^1^, ^11^ which provides more evidence for the diagnosis of CJD. However, the current RT‐QuIC technology is not useful in detecting the misfolded prions in vCJD patients. One study reported that RT‐QuIC, combined with using protein multiplication cyclic amplification (PMCA), could discriminate vCJD from other subtypes.^11^ Although the gold standard for the final diagnosis of CJD is a pathological biopsy, the procedure was not recommended for the sake of the transmissible nature of the disease and its invasiveness.

## 4 CONCLUSION

In conclusion, CJD was incurable, irreversible, and had the propensity to present atypically. It is vital to make an early and prompt diagnosis based on its clinical features due to its mortality and infectivity. In this case, the female patient's clinical manifestations and the course of the disease were consistent with the diagnostic criteria of the disease. This patient has not undergone the lumbar puncture or the brain biopsy. However, the clinical diagnosis will help the family member prepare palliative care and improve the quality of the rest life of the patient. With the advent of advanced neuroimaging, the new RT‐QuIC technology, the rate of misdiagnosis of CJD has reduced. However, it was still critical for the clinician to fully recognize this fatal neurodegenerative disease and differentiate it from other mimic diseases, especially in elderly patients, and avoid initiating inappropriate therapy.

## CONFLICT OF INTEREST

None declared.

## AUTHOR CONTRIBUTIONS

ZX: conceived and designed the study. YZ: involved in overall supervision of the paper. All authors read and approved the final manuscript.

## ETHICAL APPROVAL

This case report was approved by the ethical committee of Shanghai Jiao Tong University Affiliated Sixth People's Hospital. The written informed consent was obtained from the son of the patient for publication of this case report.

## Data Availability

The data used in this study are available from the corresponding author on reasonable request.
